# Comparison of regional and general anesthesia for retrograde intrarenal surgery: a systematic review and meta-analysis

**DOI:** 10.3389/fsurg.2025.1422660

**Published:** 2025-04-10

**Authors:** Yen Ho, Yu-Ching Wen, Liang-Ming Lee, Ke-Hsun Lin, Chi-Hao Hsiao, Syuan-Hao Syu, Benjamin Chung-Howe Lai, Cho-Hsing Chung, Yung-Wei Lin

**Affiliations:** ^1^ Department of Urology, Wan Fang Hospital, Taipei Medical University, Taipei, Taiwan; ^2^ Department of Urology, School of Medicine, College of Medicine, Taipei Medical University, Taipei, Taiwan

**Keywords:** urolithiasis, anesthesia, renal function, retrospective study, visual analog scale, retrograde intrarenal surgery, meta-analysis

## Abstract

**Objective:**

To evaluate the effectiveness and safety of retrograde intrarenal surgery (RIRS) for urolithiasis under different anesthesia methods based on current evidence.

**Materials and methods:**

In March 2022, systematic research was conducted using the databases PubMed, Embase, Google Scholar, and the Cochrane Library to compare outcomes of RIRS with regional anesthesia (RA) or general anesthesia (GA) through randomized controlled trials (RCTs) and observational studies. Data analysis was performed using the comprehensive meta-analysis software version 3.

**Results:**

Eight RCTs and one retrospective study, involving a total of 2,111 individuals, were included in the current review. Pooled data revealed no significant statistical differences in RIRS under RA compared to RIRS under GA in terms of stone-free rate (SFR) [odds ratio (OR) = 1.02, *p* = 0.94], operating duration [weighted mean difference (MD) = −0.04, *p* = 0.88], length of hospital stay (MD = −0.05, *p* = 0.63), postoperative first-day visual analog scale score (MD = 0.18, *p* = 0.30), and complication rates (OR = 0.83, *p* = 0.20). However, one of the RCTs reviewed showed a potential negative effect of GA on the renal function at the operative site. Maneuverability and accessibility were found to be significantly better with SA and sedation than with GA. Additionally, the cost of GA was noted to be significantly higher than that of RA, according to more than one RCT.

**Conclusion:**

The present study revealed that RIRS under RA is not inferior in effectiveness and safety compared to that under GA, in terms of SFR, operating time, length of hospital stay, postoperative pain scores, and complication rates. Moreover, RA may offer better long-term renal function preservation and be more cost effective than GA. To improve maneuverability and accessibility for operators, we suggested that RA with sedation could be a suitable alternative with careful patient selection.

**Systematic Review Registration:** https://www.crd.york.ac.uk/prospero/display_record.php?ID=CRD42023463411, identifier: CRD42023463411.

## 1 Introduction

Urolithiasis is a common urological disorder with varying prevalence rates across different geographic regions. Specifically, the prevalence rate ranges from 7%–13% in North America, from 5%–9% in Europe, and from 1%–5% in Asian regions (1, 2). According to the urolithiasis guidelines of the European Association of Urology, retrograde intrarenal surgery (RIRS) is recommended as a primary therapeutic option, along with shockwave lithotripsy, for renal calculi smaller than 2 centimeters in diameter (3).

General anesthesia (GA) has traditionally been used for conducting RIRS. While there are no absolute contraindications to GA, except when the patient explicitly declines it, there are several relative contraindications to consider. These include difficult airway access and the presence of substantial comorbidities such as severe aortic stenosis, pulmonary diseases, and congestive heart failure. These factors may contribute to patient reluctance, especially for elective surgery (4).

In contrast, regional anesthesia (RA) has emerged as an alternative. It is important to differentiate between RA and local anesthesia (LA) since they are sometimes confused. While LA numbs a small, localized area for minor procedures, RA involves blocking nerve pathways to anesthetize larger regions of the body, such as with spinal or epidural anesthesia (5). In the context of RIRS, RA—particularly spinal or epidural anesthesia—has been increasingly used, especially in patients for whom GA poses higher risks, or in settings where cost and resource constraints are important considerations.

Previous studies have demonstrated that patients undergoing percutaneous nephrolithotomy RA have lower morbidity and mortality rates compared to GA (6, 7). Recent research has focused on the feasibility and safety of RIRS with RA, revealing that RA can provide comparable efficacy and safety outcomes as GA (8–10). While several studies have investigated potential predictive factors influencing RIRS outcomes, the consensus on the significance of anesthesia type remains unclear. This study aimed to compare the success and complication rates of RIRS conducted under RA and GA. We hypothesized that RA could be a safe and potentially superior anesthesia option for RIRS, specifically for renal function preservation and cost-effectiveness.

## 2 Materials and methods

### 2.1 Search strategy

A comprehensive and systematic literature search was conducted across various reputable databases, including PubMed, Embase, Google Scholar, and the Cochrane Library. We registered the study protocol on PROSPERO (No CRD42023463411) prospectively. Moreover, the search employed specific keywords such as “flexible ureteroscopy,” “retrograde intrarenal surgery,” “RIRS,” “anesthesia.” These search terms were adjusted as needed for each database, and MeSH terms were integrated. Additional studies were identified through the references of relevant articles. Details of the literature search for all databases are presented in Supplementary Table S1. The language criteria included articles in English or Chinese. The search process adhered to the guidelines outlined by the preferred reporting items for systematic reviews and meta-analysis (PRISMA) (11) and assessing the methodological quality of systematic reviews (AMSTAR).

### 2.2 Inclusion and exclusion criteria

The eligible studies were selected based on specific inclusion and exclusion criteria following the PICOS (population, intervention, comparison, outcome, and study design) framework. The inclusion criteria included:

- Population: adults (aged over 18 years) diagnosed with upper urinary tract stones undergoing RIRS
- Intervention: patients undergoing RIRS with RA (spinal anesthesia, SA or combined spinal-epidural anesthesia, CSEA)
- Comparison: patients undergoing RIRS under GA
- Outcomes: stone-free rate (SFR), operation time, length of hospital stay, pain scores, complications
- Study design: randomized controlled trials (RCTs), nonrandomized controlled trials (NRCTs) such as controlled clinical trials, observational studies

For randomized controlled trials (RCTs), there was no requirement for a specific minimum sample size. However, for nonrandomized controlled trials (NRCTs), a minimum of 200 participants per study arm was mandated to ensure sufficient statistical power and to reduce the influence of potential confounding factors associated with nonrandomized study designs.

Conversely, studies were excluded if they included patients with contraindications for GA or RA, such as severe respiratory conditions or coagulopathies. Additionally, studies were excluded if they included outcomes that could not be analyzed or if they were published in the form of reviews, case reports, or commentary pieces.

### 2.3 Data extraction

Data from the selected articles were independently extracted by two reviewers using a predefined extraction form. The following items were extracted: first author, year of publication, country, study design, intervention, sample size, SFR, operation duration, length of hospital stay, postoperative visual analog scale (VAS) scores, and overall complications. Any discrepancies were resolved through consultation with a third reviewer.

### 2.4 Quality assessment

Two investigators independently assessed the methodological quality and levels of evidence for all relevant clinical studies. Any discrepancies in the evaluation were resolved through discussion. The Oxford Centre for Evidence-Based Medicine guidelines (12) were employed to determine the level of evidence for each study. Methodological quality appraisal for eligible case-control trials was assessed using the Newcastle–Ottawa Scale (13). The total score ranges from 0–8, with a score >5 indicating an acceptable methodological design. The Jadad scale (14) was used to assess the methodological quality of the included RCTs. We defined the studies with scores of 3–5 as high quality, and those with scores of 0–2 as low quality. The risk-of-bias in RCTs was evaluated using the Cochrane Handbook Risk-of-bias table (15).

### 2.5 Statistical analysis

The statistical analyses were conducted using comprehensive meta-analysis software version 3. The *χ*^2^ and *I*^2^ tests were used to assess the heterogeneity of the study data. An *I*^2^ value exceeding 50% corresponded to high heterogeneity, and the random-effects models was selected for the meta-analyses. Low heterogeneity was identified and a fixed-effects model was applied. Pooled relative risks or odds ratios, along with their respective 95% confidence intervals, were computed for categorical outcomes. For continuous outcomes, pooled mean differences and their corresponding 95% confidence intervals were calculated. Statistical significance for hypothesis testing was set at a two-tailed alpha level of 0.05. Throughout the analysis, unadjusted *p*-values were reported. Additionally, the funnel plot was used to assess potential publication bias.

## 3 Results

### 3.1 Characteristics and quality

**Figure 1:**
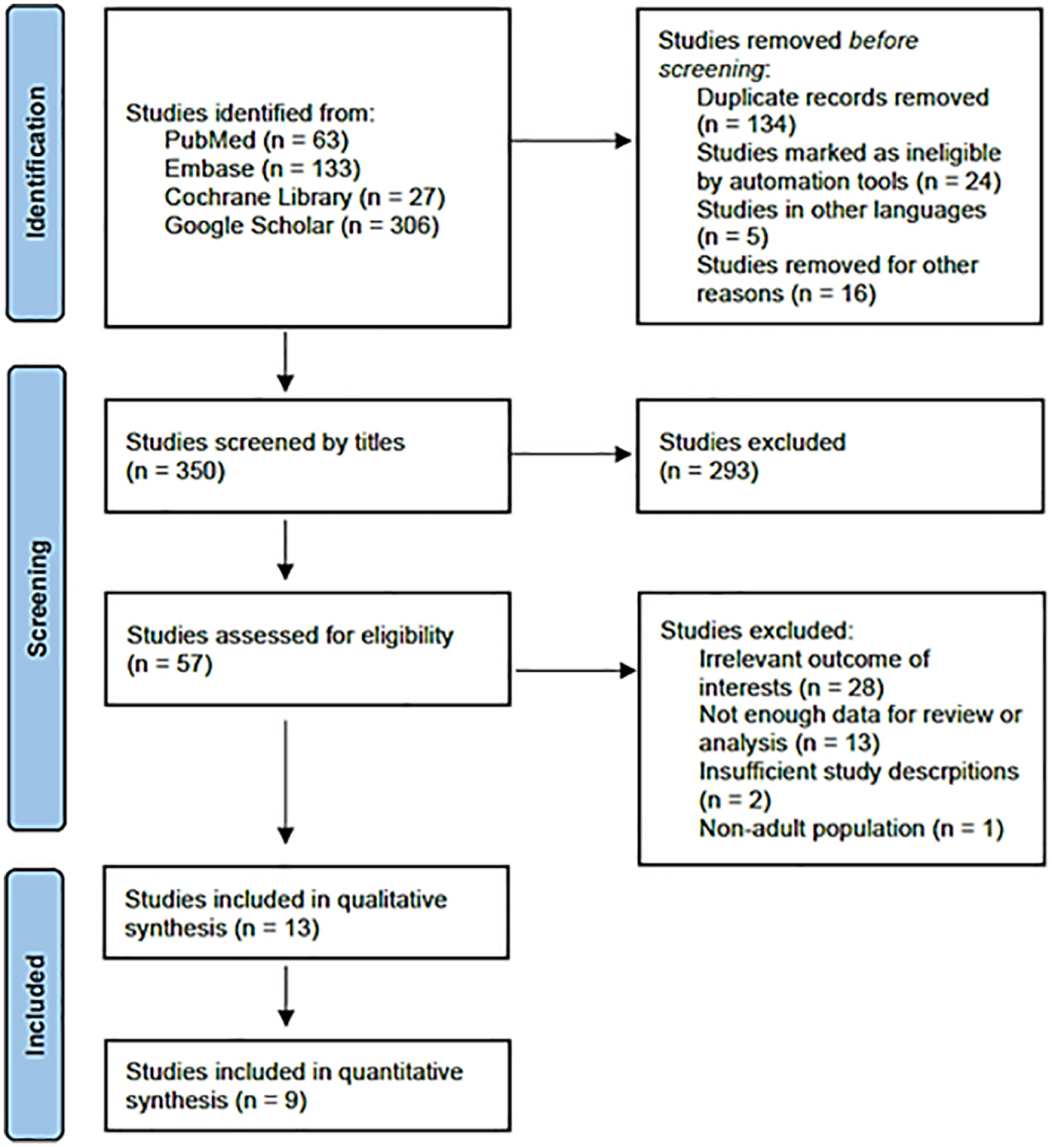
PRISMA flow diagram of study selection process.

Based on the literature search and inclusion criteria, 350 studies were initially identified. Ultimately, 13 studies were selected for analysis, comprising 1,049 cases of RA and 1,062 cases of GA. A detailed flowchart of the literature selection process is presented in Figure 1. Nine studies were included for data analysis, with one of them not being an RCT (9, 16–23). Characteristics and outcome summaries of the included studies are presented in Table 1. The authors' judgments on the risk-of-bias in the studies are shown in Supplementary Figure S1.

**Table 1:** Summary of comparative studies included in the meta-analysis.

Study	Country	Study design	Intervention	Sample size	Mean age (years)	Gender (M/F)	Mean stone size (mm)	Mean stone density (HU)	Outcomes summary	LE	Study quality
Çakici et al. (16)	Turkey	RCT	CSEA	45	46.7 ± 14.6	26/19	16.1 ± 5.3	779.9 ± 175.9	No significant difference in SFR, OT, LOS, VAS score or complication rates.	2a	4
			GA	50	42.8 ± 11.4	31/19	13.9 ± 7	764 ± 164.9			
Karabulut et al.(17)	Turkey	RCT	SA	43	NR	NR	NR	NR	VAS score: SA < GA; no significant difference in SFR, OT, LOS or complication rates.	2a	3
			GA	43							
Kwon et al. (18)	Korea	RCT	SA	31	54.7 ± 14	20/11	12 ± 3.4	961.1 ± 354.4	SFR: SA < GA; VAS score: SA < GA^b^; no significant difference in OT, LOS or complication rates.	2a	4
			GA	39	54.1 ± 14.5	26/13	11.3 ± 3.3	937 ± 294.2			
Li et al. (19)	China	RCT	CSEA	89	31.2 ± 9	39/50	NA^a^	NR	SFR: CSEA < GA^c^; OT: CSEA > GA; cost: CSEA < GA.	2a	3
			GA	105	36.3 ± 7.9	63/42					
Oztekin et al. (20)	Turkey	RCT	SA	35	45.8 ± 15.4	25/10	12.7 ± 3.6	1035.8 ± 371.8	Stone access time: SA > GA; no significant difference in SFR, OT, VAS score or complication rates.	2a	4
			GA	35	44.9 ± 14.6	23/12	13 ± 3.8	1,116 ± 294.9			
Zeng et al. (21)	China	RCT	CSEA	31	47.6 ± 11.6	20/11	19 ± 9	847.6 ± 295.2	Hb drop: CSEA < GA; cost: CSEA < GA; no significant difference in SFR, OT, LOS, VAS score or complication rates.	2a	4
			GA	34	49.3 ± 11.3	20/14	24 ± 13	811.8 ± 294.7			
Baran et al.(9)	Turkey	CCT	SA	697	47 ± 14.2	479/218	17.6 ± 5.9	779.9 ± 175.9	OT: SA < GA; no significant difference in SFR, LOS, VAS score or complication rates.	3b	NA^d^
			GA	664	48.4 ± 14	434/230	17.2 ± 6	764 ± 164.9			
Sahan et al. (22)	Turkey	RCT	CSEA	45	44.1 ± 12.6	26/19	15.7 ± 7.3	NR	Complication rates: CSEA > GA; surgeon comfort: CSEA < GA; no significant difference in SFR, OT, LOS or VAS score.	2a	4
			GA	61	46 ± 16.3	35/26	17.2 ± 7.7				
Mohamed et al. (23)	Saudi Arabia	RCT	SA	33	NR	NR	NR	NR	LOS: SA < GA; no significant difference in SFR, OT, VAS score or complication rates.	2a	4
			GA	31							

HU, hounsfield units; LE, level of evidence; RCT, randomized controlled trial; CSEA, combined spinal-epidural anesthesia; SA, spinal anesthesia; GA, general anesthesia; NR, not reported; M, male; F, female; SFR, stone-free rate; OT, operation time; LOS, length of stay; VAS, visual analog scale; Hb, hemoglobin.

^a^ Li et al. estimated stone sizes in terms of mean stone burden: CSEA, 161 mm^2^; GA, 165 mm^2^.

^b^ VAS scores are significant lower for the SA group than GA group on the morning of the first postoperative day (*p* = 0.025), but not immediately after the operation (*p* = 0.178) or before discharge (*p* = 0.560).

^c^ Significant difference only in SFR estimated 3 months postoperatively (*p* = 0.049), but not in SFR estimated 1 day after the operation (*p* = 0.066).

^d^ The Newcastle–Ottawa Scale (NOS) score is 6.

### 3.2 Stone-free rate

**Figure 2:**
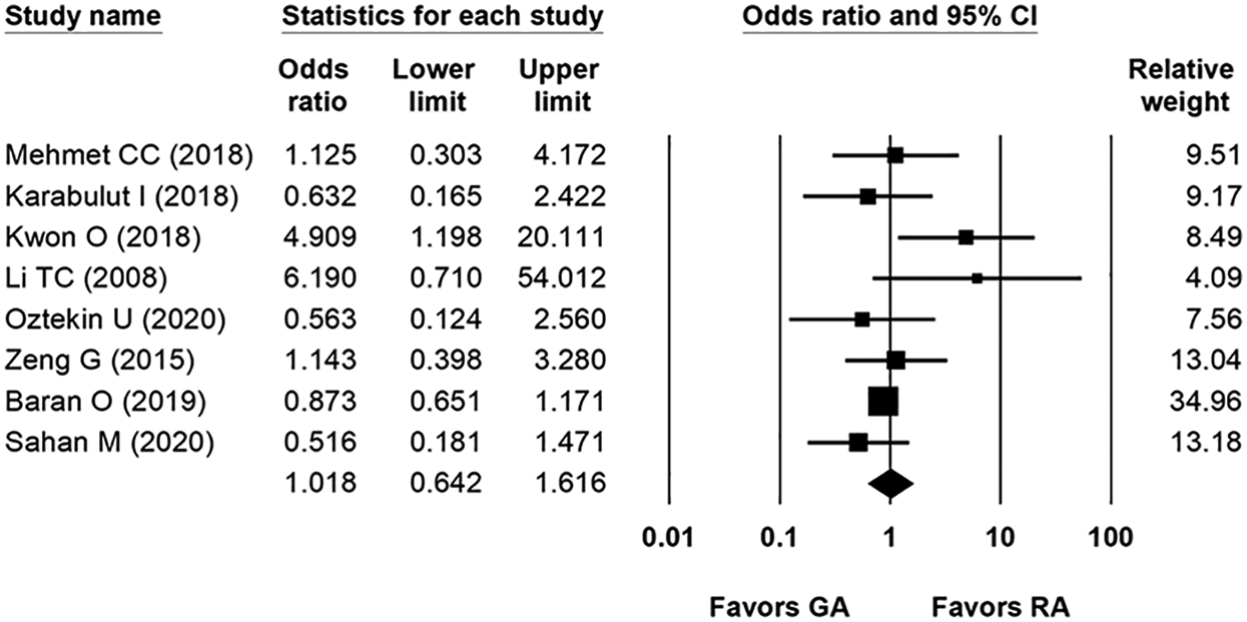
Forest plot comparing stone-free rate between general anesthesia and regional anesthesia groups.

Eight studies were included in the forest plot for the SFR. SFR was defined as complete stone clearance or a maximum residual fragment smaller than 2 mm. Postoperative assessments (Kidneys-Ureters-Bladder radiography, ultrasound, and abdominal computed tomography scan) were conducted 1–3 months after the operation. The overall SFRs in the included studies were 85.2% (878/1,031) for GA and 85.8% (872/1,016) for RA. The heterogeneity was low (*p* = 0.1155, *I*^2^ = 34%). There was no evidence of publication bias on visual inspection of the funnel plot (Supplementary Figure S2). The fixed-effects model indicated that the two groups were statistically comparable in terms of SFR [odds ratio (OR): 1.018, 95% CI: 0.642–1.616, *p* = 0.518; Figure 2]. Additionally, subgroup analysis was performed according to different RA types and showed similar results between CSEA and SA compared to GA (Supplementary Figure S3).

### 3.3 Operation/lithotripsy time

**Figure 3:**
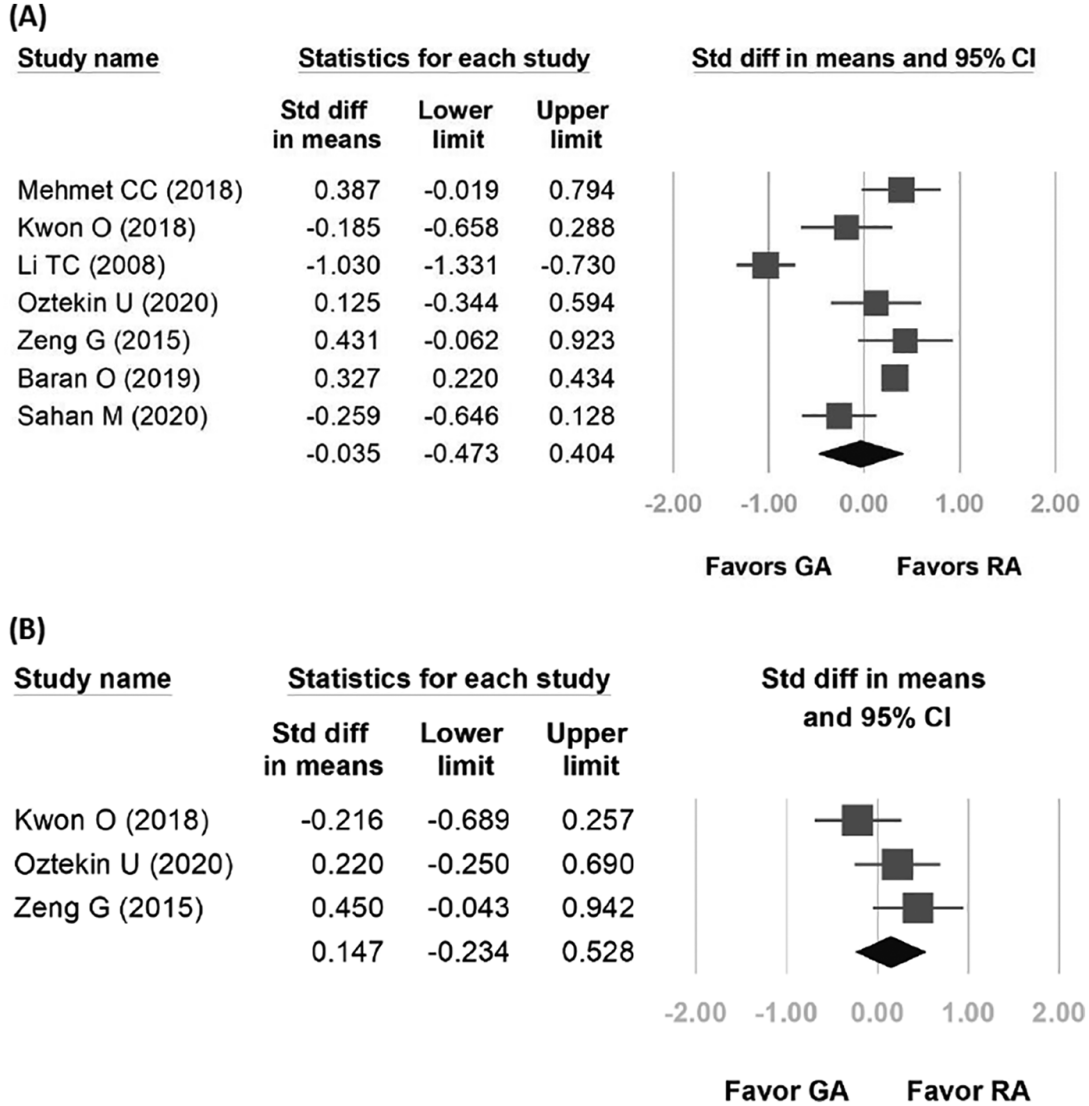
Forest plot comparing operative time and lithotripsy time between general anesthesia and regional anesthesia groups. **(A)** Operative time; **(B)** Lithotripsy time.

Seven studies were included in the forest plot for operative time. The mean operation time ranged from 44.92–57.6 min for GA and 39.33–64 min for RA. Despite high heterogeneity (*p* < 0.001, *I*^2^ = 92.165%), the fixed-effects model indicated no statistical difference between the two groups [standardized difference (SD): −0.035, 95% CI: −0.473, 0.404, *p* = 0.876, Figure 3A]. Subgroup analysis of the operations duration according to different RA types was performed, which revealed similar results between CSEA and SA comparing to GA. (Supplementary Figure S4) Only three studies were included in the forest plot for lithotripsy time. The lithotripsy time ranged from 15.6–38.7 min for GA and 19–29.9 min for RA. The heterogeneity was moderate (*p* = 0.149, *I*^2^ = 47.41%), and the fixed-effects model indicated that the two groups were statistically comparable (SD: 0.147, 95% CI: −0.234, 0.528, *p* = 0.449; Figure 3B).

### 3.4 Length of stay

**Figure 4:**
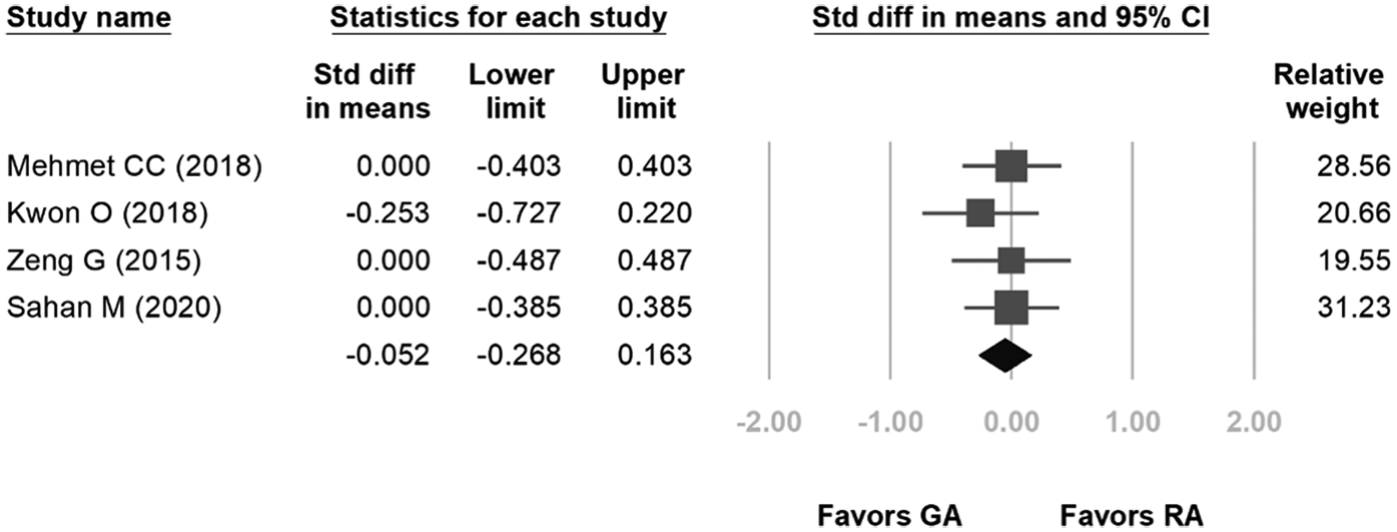
Forest plot comparing postoperative pain scores.

Four studies were included in the forest plot for length of stay. The length of hospital stay ranged from 1.07–1.9 days for GA and 1.06–1.9 days for RA. The heterogeneity was low (*p* = 0.832, *I*^2^ = 0%), and the fixed-effects model indicated no statistical difference between the two groups in terms of length of hospital stay (SD: −0.052, 95% CI: −0.268, 0.163, *p* = 0.634, Figure 4).

### 3.5 Complications

**Figure 5:**
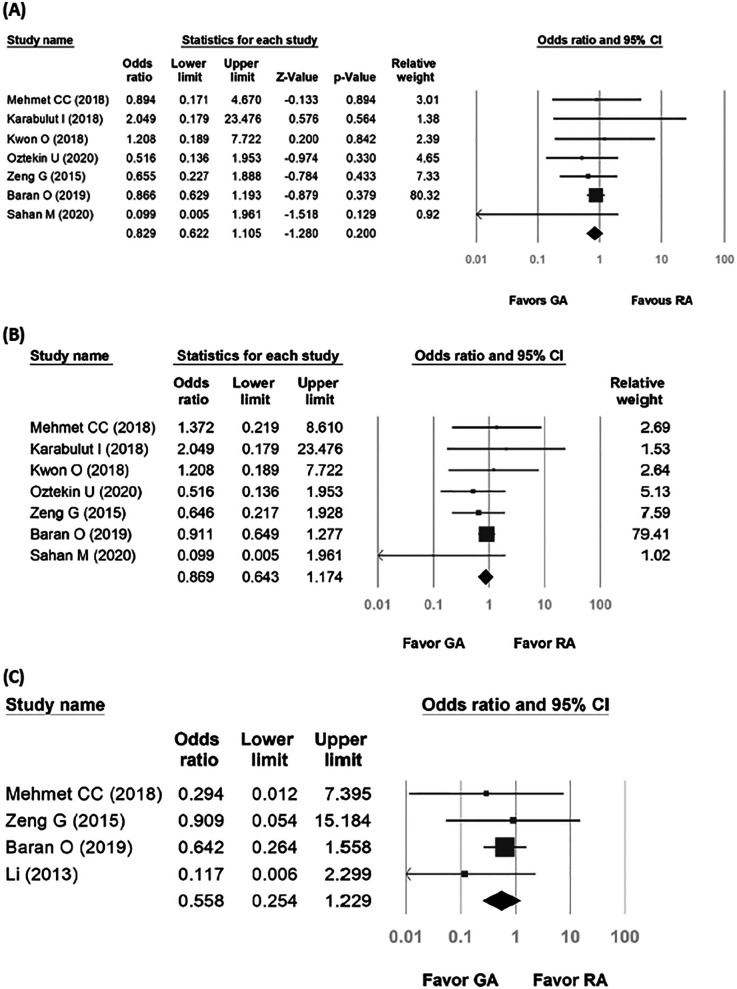
Forest plot comparing complication rates between general anesthesia and regional anesthesia groups. **(A)** Total complication rates; **(B)** Grade I and II complication rates; **(C)** Grade III and above complications.

Seven studies were included in the forest plot for total complication rates. The heterogeneity was low (*p* = 0.778, *I*^2^ = 0%), and the fixed-effects model indicated no statistical difference between the two groups in terms of total complication rates (OR: 0.829, 95% CI: 0.622–1.105, *p* = 0.200, Figure 5A). Upon visual examination of the funnel plot, no indication of publication bias was noted (Supplementary Figure S5). For mild complications, seven studies were included in the forest plot of Clavien–Dindo Grade I and II complication rates. The heterogeneity was low (*p* = 0.702, *I*^2^ = 0%), and the fixed-effects model indicated no statistical difference between the two groups in terms of mild complication rates (OR: 0.869, 95% CI: 0.643–1.174, *p* = 0.360, Figure 5B). For moderate or severe complications, four studies were included in the forest plot of Clavien–Dindo Grade III and above complication rates. The heterogeneity was low (*p* = 0.901, *I*^2^ = 0%), and the fixed-effects model indicated no statistical difference between the two groups in terms of moderate or severe complication rates (OR: 0.558, 95% CI: 0.254–1.229, *p* = 0.148; Figure 5C).

### 3.6 Postoperative 24 h visual analog scale score

**Figure 6:**
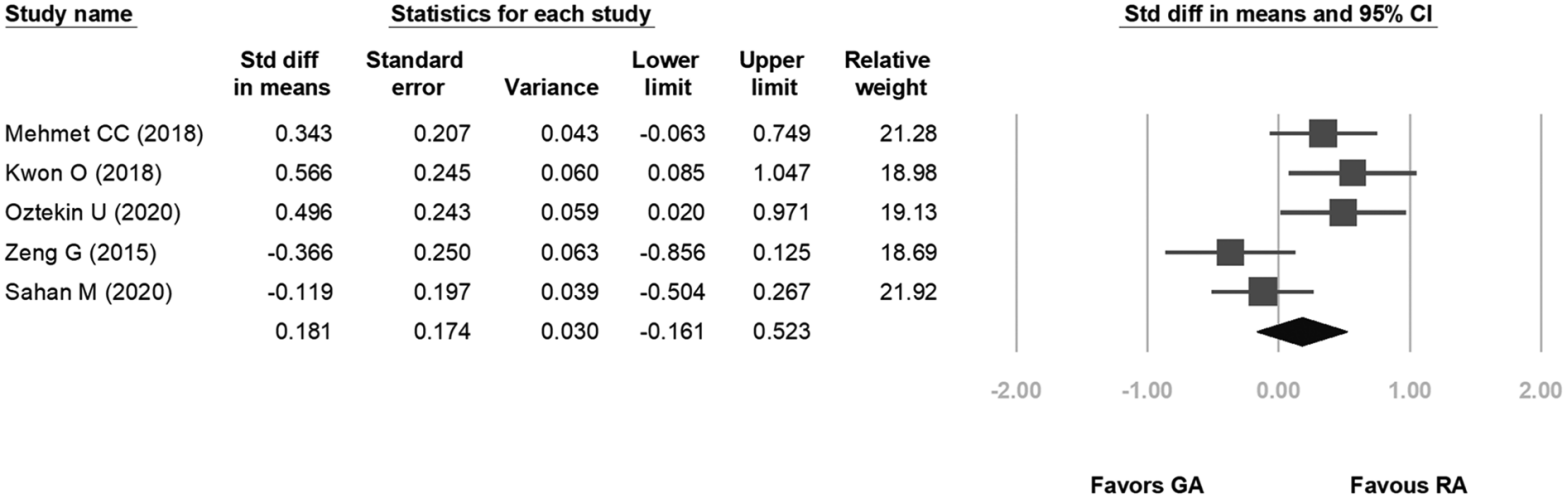
Forest plot comparing length of hospital stay.

Five studies were included in the forest plot for 24-hour postoperative pain. The VAS scores ranged from 0.7–4.9 for GA and 0.8–3.7 for RA. The heterogeneity was low (*p* = 0.019, *I*^2^ = 66.201%), and the fixed-effects model indicated no statistical difference between the two groups in terms of postoperative pain scores (SD: 0.181, 95% CI: −0.161, 0.523, *p* = 0.30; Figure 6).

## 4 Discussion

RIRS has emerged as a primary approach for treating upper urinary tract stones, due to its minimally invasive nature. The European Association of Urology guidelines recommend GA as the preferred choice for this procedure, with RA occasionally used (1). In contrast, the American Urological Association guidelines do not provide specific recommendations on anesthesia selection. There is no official consensus on the optimal anesthesia type for RIRS, as both GA and RA have their own advantages and disadvantages in terms of surgical success, complication rates, and patient and surgeon preferences. For patients with compromised cardiopulmonary function or multiple comorbidities, opting for GA over RA may increase the risk of complications related to endotracheal intubation. However, GA can sometimes offer better intraoperative patient management. Several meta-analyses have suggested the benefits of using RA for percutaneous nephrolithotomy, including shorter operating times and reduced postoperative discomfort (24–26). Therefore, assessing the impact of anesthesia type on RIRS outcomes is important. Our meta-analysis indicates that using RA in RIRS is comparable to GA in terms of surgical and anesthesia efficacy and safety. There were no significant differences in SFRs, operating durations, length of hospital stay, or complication rates between RA and GA for RIRS. This finding enables surgeons and anesthesiologists to make more informed decisions on the most appropriate anesthesia method based on individual patient factors.

The SFR serves as a pivotal indicator of success in urological stone surgeries. Traditionally, urologists have preferred conducting RIRS under GA due to better control over diaphragmatic movements and tidal volume, which are believed to impact surgical outcomes. GA enables machine-controlled breathing regulation and adjustments to tidal volume to manage breathing patterns and posture, reducing kidney stone movement (27, 28). Conversely, patients under RA, especially those maintaining spontaneous breathing, may pose challenges for urologists in targeting the laser during lithotripsy in mobile kidneys. However, recent studies suggest that this can be managed by instructing patients to momentarily hold their breath during critical laser lithotripsy phases, mimicking the effect of induced apnea under GA. This technique has been used successfully in other studies and may help improve procedural precision (29). Furthermore, patients' fear or anxiety during the operation can hinder cooperation, particularly in cases with language barriers or limited self-control. Furthermore, patients under RA are awake, and changing their positions can cause discomfort or even lead to the discontinuation of the procedure (30). However, our findings revealed no significant difference in SFR between the GA and RA groups, suggesting that kidney motility may not significantly impact procedural success. Although our results align with most studies, Kwon *et al*. reported a higher SFR in the GA group than in the RA group (71.0% *vs*. 92.3%, *p* < 0.05). They administered sedation during RA using 1–3 mg of midazolam, which was believed to enhance operability and accessibility, potentially surpassing GA outcomes. Although direct evidence is lacking, the benefits of enhanced operability and accessibility with sedation may positively impact SFR, making RA with sedation a viable alternative to GA (18). It is important to acknowledge the variability in SFR definitions and follow-up durations across studies. Future investigations for a standardized definition of SFR and follow-up duration are required to ensure consistency in reporting and comparison of outcomes.

The operative time is crucial in preventing postoperative complications, particularly in cases of ureteral perforation and subsequent urinary tract infections. Our meta-analysis revealed no significant differences in the operative time or stone fragmentation time between patients undergoing RIRS under RA and those under GA. However, there was considerable heterogeneity in these findings. While one study showed a notably shorter operative time in the GA group (19), other studies either reported no substantial differences or shorter operative and stone fragmentation times in the RA group. Moreover, the variability in operative time may be attributed to various factors, such as the inclusion of anesthesia time within the recorded operative time, which may vary based on individual anatomical characteristics. Surgeon experience and stone characteristics can also influence the operative time. Among the studies reviewed, only Kwon *et al*. assessed operator maneuverability and accessibility, demonstrating better performance under spinal anesthesia with sedation compared to GA (18). Although there is no robust clinical evidence linking enhanced maneuverability to substantially increased SFRs or reduced operative time, spinal anesthesia with sedation remains a viable and favorable alternative to GA. Nevertheless, Olivero *et al*. reported a higher incidence of interrupted procedures in the RA group due to challenges in reaching the renal pelvis with the instruments. Although the difference (10% vs. 2.5%) was not statistically significant (*p* = 0.166), which suggests potential difficulties with this approach (10).

Among the studies reviewed, Kwon *et al*. are the only ones to explore the comparative effects of the two anesthesia methods on renal function (18). Although the majority of studies have focused on non-urologic surgeries, they have underlined GA as a potential risk factor for postoperative renal dysfunction. A comprehensive review of randomized trials also suggested that RA may reduce the incidence of serious postoperative complications, including overall mortality rates, cardiopulmonary complications, and incidents of renal failure following surgeries conducted under RA (7). For instance, Hassan *et al*. reported changes in renal function after total hip replacement procedures, where GA emerged as a significant risk factor for elevated serum creatinine levels (*p* = 0.0083). The same researchers also identified GA as a risk factor for postoperative renal dysfunction following total knee replacement surgeries (31). According to Suleiman *et al*., the impact of epidural anesthesia on renal blood flow has been found to be statistically insignificant (32). Moreover, a subsequent meta-analysis has shown that when combined with GA, epidural anesthesia has a mitigating effect on the occurrence of perioperative acute renal failure, particularly in cardiac surgery patients (33). A thorough review has examined the potential of bupivacaine, a medication commonly used in RA, to alleviate cellular damage, suggesting its potential protective capacity in ameliorating renal ischemia/reperfusion injury (34). However, clinicians should acknowledge the existing discrepancy in evidence regarding the influence of anesthetics on renal function.

While RIRS is known for its minimal invasiveness from a patient perspective, the duration of the procedure is a significant concern due to its association with postoperative complications, particularly ureteral perforations (35). Our comprehensive analysis revealed no significant differences in the operative time or stone fragmentation time between patients undergoing RIRS under RA and GA. However, there was a notable degree of heterogeneity in the results, particularly within the combined spinal and epidural anesthesia subgroup. Upon exclusion of the study conducted by Li et al. (19), the sources of heterogeneity were mitigated, and the findings pointed to an improvement in operative time with RA. This finding may be attributed to the inclusion of anesthesia time within the operative time in the study. It is worth noting that RA's unique characteristics, such as the determination of anesthesia block, can result in longer anesthesia times compared to GA, distinguishing it from findings in similar studies (24). Additionally, surgeon experience and individual patient characteristics also influence the procedure duration, with a higher stone burden typically contributing to longer operative and lithotripsy times (36). Given the inherent limitations of the trials included in our analysis, future studies should adhere to rigorous standards to yield more reliable and consistent outcomes.

Different anesthesia methods are associated with different sets of complications. RA carries a higher risk of hypotension and headaches, due to its sympathetic effects and the potential for dural perforation (16). In contrast, GA is often linked to complications involving the vascular, pulmonary, and neurological systems (37). However, our analysis revealed no substantial differences in the incidence of Clavien–Dindo Grade I, II, and III complications between the RA and GA groups. This trend was also applied to postoperative symptoms like fever, nausea, and discomfort. While RIRS is typically associated with minor complications, critical complications like kidney injury, acute sepsis, ureter avulsion, and arteriovenous fistula have been reported in the literature (38). Studies by Lildal et al. (39) and Mao et al. (40) explored ureteral injuries in RIRS, highlighting the impact of ureteral access sheath (UAS) use and insertion resistance. While neither study examined anesthesia type, their findings on mechanical trauma risks remain relevant for endourologists concerned about patient movement under RA. Given the theoretical risk, preoperative counseling, intraoperative patient communication, and potential mild sedation may help reduce unintended movements during critical phases of the procedure. Notably, the studies included in this analysis did not report any other severe complications. Therefore, RA appears to offer a comparable level of safety to GA. Nevertheless, due to the restricted number of studies and relatively modest sample sizes for assessing various complications, further research with larger sample sizes is needed to fully assess the safety profile of RA.

This study has certain limitations that should be considered. First, the patient cohort was heterogeneous, with the non-RCT study including a significantly larger population. This disparity could potentially impact some of the quantitative findings. However, the implementation of a minimum sample size criterion for NRCTs ensures that only studies with sufficient statistical power were included. This approach helped to mitigate some of the inherent biases in nonrandomized designs, thereby enhancing the overall reliability of our findings. Second, the cost-effectiveness of the two anesthesia approaches was not assessed in this study, which should be explored in future research. Lastly, relevant factors, such as stone sizes, laterality, and density, were not assessed, which could affect outcomes. Therefore, it is important to interpret the study findings with caution due to the aforementioned limitations.

## 5 Conclusion

Our study demonstrates that RIRS conducted under RA is not inferior to procedures under GA in terms of effectiveness and safety. RA may also offer advantages in terms of long-term preservation of renal function and economic considerations compared to GA. To enhance operator maneuverability and accessibility, we recommend that RA with sedation could be a suitable alternative, depending on careful patient selection.

## Data Availability

The original contributions presented in the study are included in the article/Supplementary Material, further inquiries can be directed to the corresponding author.
