# Supplementary material for: Comparison of regional and general anesthesia for retrograde intrarenal surgery: a systematic review and meta-analysis
Source: Front Surg. 2025 Apr 10;12:1422660. doi: 10.3389/fsurg.2025.1422660 (PMC12018349; doi:10.3389/fsurg.2025.1422660)
Supplement: Supplementary file 1 [file Table1.docx]

Supplementary Material

# Supplementary Table

Supplementary Table 1. Search strategy

| Database | Query | Search Details | Results |
| --- | --- | --- | --- |
| PubMed | (retrograde intrarenal surgery) AND (anesthesia) | ("retrograde"[All Fields] OR "retrogradely"[All Fields]) AND ("intrarenal"[All Fields] OR "intrarenally"[All Fields]) AND ("surgery"[MeSH Subheading] OR "surgery"[All Fields] OR "surgical procedures, operative"[MeSH Terms] OR ("surgical"[All Fields] AND "procedures"[All Fields] AND "operative"[All Fields]) OR "operative surgical procedures"[All Fields] OR "general surgery"[MeSH Terms] OR ("general"[All Fields] AND "surgery"[All Fields]) OR "general surgery"[All Fields] OR "surgery s"[All Fields] OR "surgerys"[All Fields] OR "surgeries"[All Fields]) AND ("anaesthesia"[All Fields] OR "anesthesia"[MeSH Terms] OR "anesthesia"[All Fields] OR "anaesthesias"[All Fields] OR "anesthesias"[All Fields]) | 72 |
|  | (RIRS) AND (anesthesia) | "RIRS"[All Fields] AND ("anaesthesia"[All Fields] OR "anesthesia"[MeSH Terms] OR "anesthesia"[All Fields] OR "anaesthesias"[All Fields] OR "anesthesias"[All Fields]) | 57 |
|  | (Flexible ureteroscopy) OR (Flexible ureterorenoscopy) AND (anesthesia) | ((("flexibilities"[All Fields] OR "flexible"[All Fields] OR "flexibles"[All Fields] OR "pliability"[MeSH Terms] OR "pliability"[All Fields] OR "flexibility"[All Fields]) AND ("ureteroscopy"[MeSH Terms] OR "ureteroscopy"[All Fields] OR "ureteroscopies"[All Fields])) OR (("flexibilities"[All Fields] OR "flexible"[All Fields] OR "flexibles"[All Fields] OR "pliability"[MeSH Terms] OR "pliability"[All Fields] OR "flexibility"[All Fields]) AND ("ureterorenoscopies"[All Fields] OR "ureterorenoscopy"[All Fields]))) AND ("anaesthesia"[All Fields] OR "anesthesia"[MeSH Terms] OR "anesthesia"[All Fields] OR "anaesthesias"[All Fields] OR "anesthesias"[All Fields]) | 88 |
| Embase | retrograde AND intrarenal AND surgery AND anesthesia | Sources: Embase, MEDLINE, Preprints  Mapped terms: n/a | 207 |
|  | rirs AND anesthesia | Sources: Embase, MEDLINE, Preprints  Mapped terms: n/a | 184 |
|  | (flexible AND ureteroscopy OR (flexible AND ureterorenoscopy)) AND anesthesia | Sources: Embase, MEDLINE, Preprints  Mapped terms: n/a | 313 |
| Cochrane Library | (retrograde intrarenal surgery) AND (anesthesia) | nil | 49 |
|  | (RIRS) AND (anesthesia) | nil | 44 |
|  | (Flexible ureteroscopy) OR (Flexible ureterorenoscopy) AND (anesthesia) | nil | 285 |

**2 Supplementary Figures**


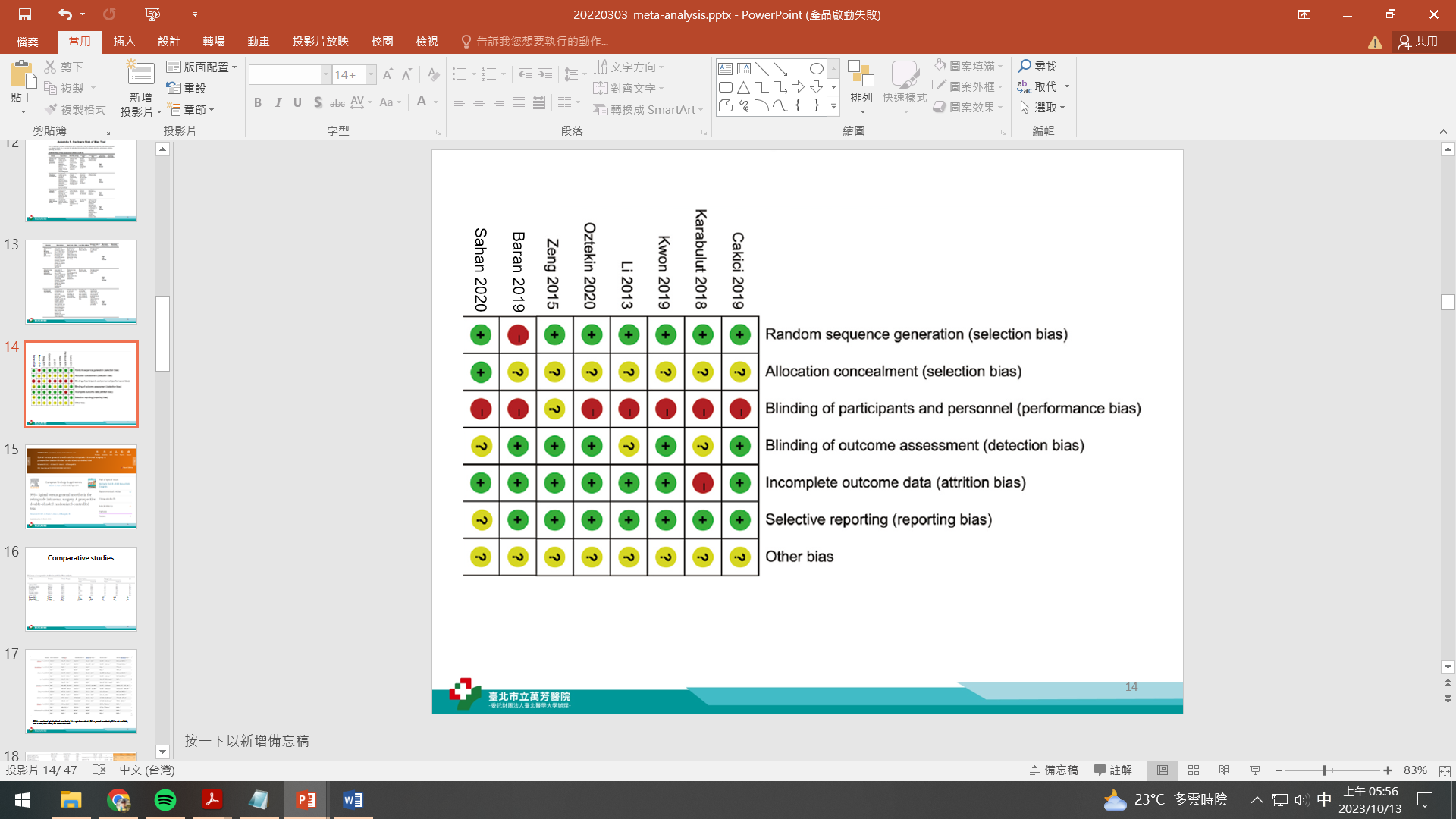


**Supplementary Figure 1.** Risk-of-bias assessed using the Cochrane Collaboration tool


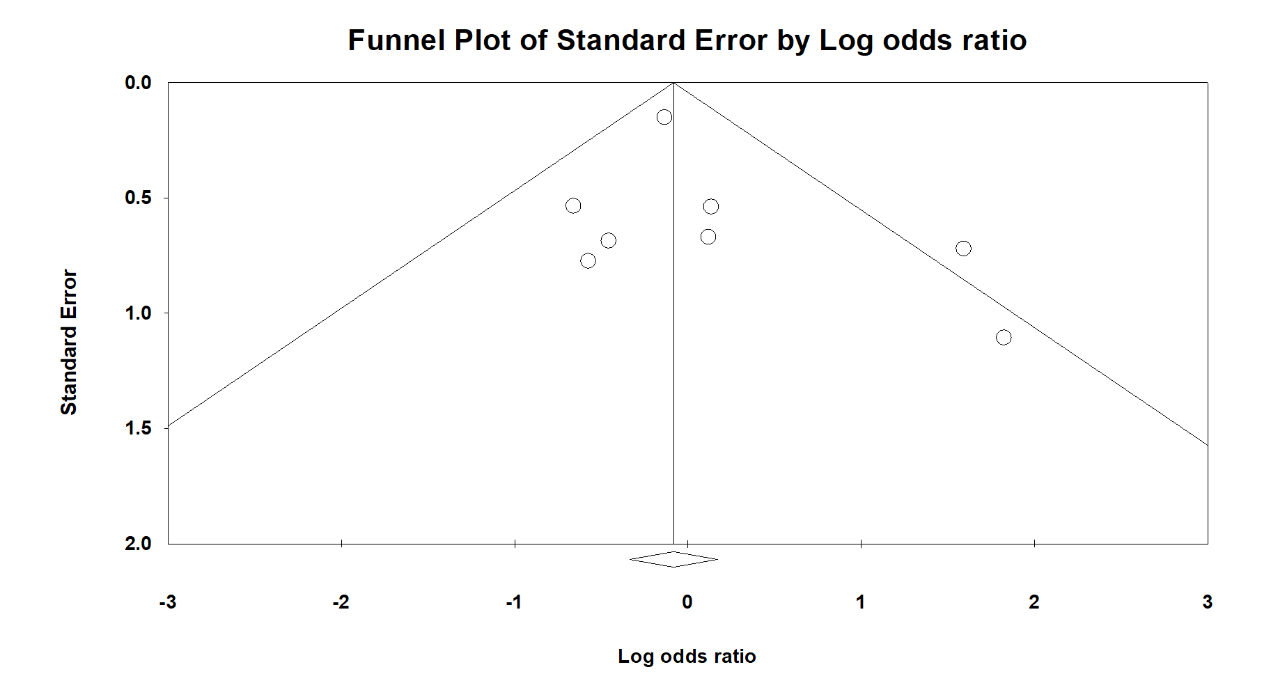


**Supplementary Figure 2.** Funnel plot for the meta-analysis of stone-free rates between RA and GA


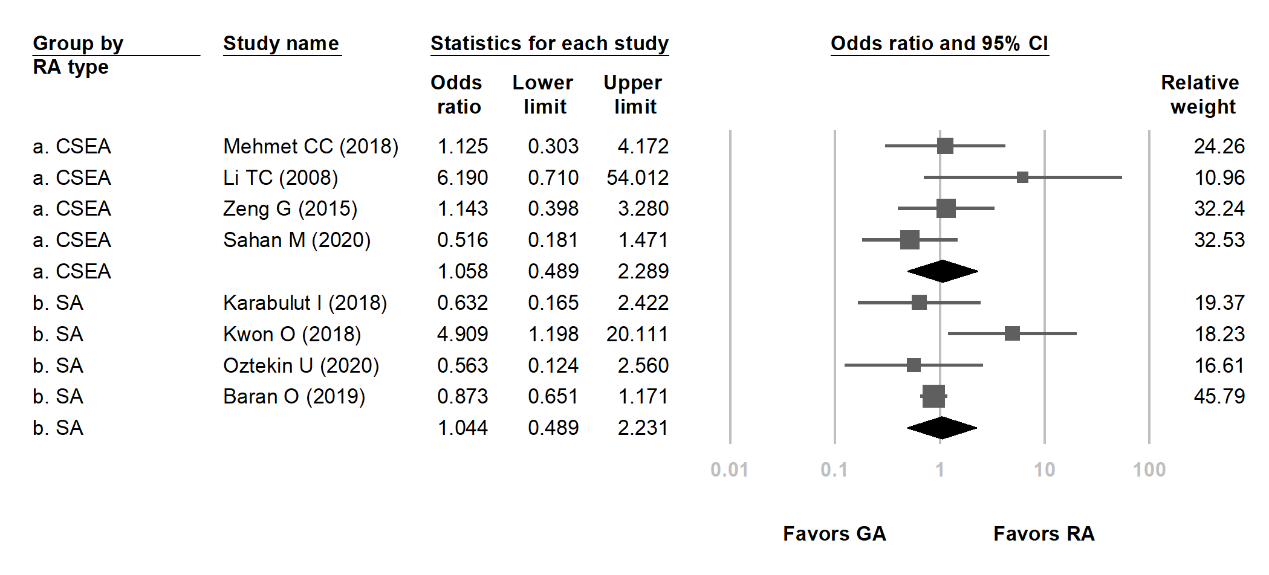


**Supplementary Figure 3.** Subgroup analysis forest plots for the stone-free rate according to RA types (CSEA vs SA)


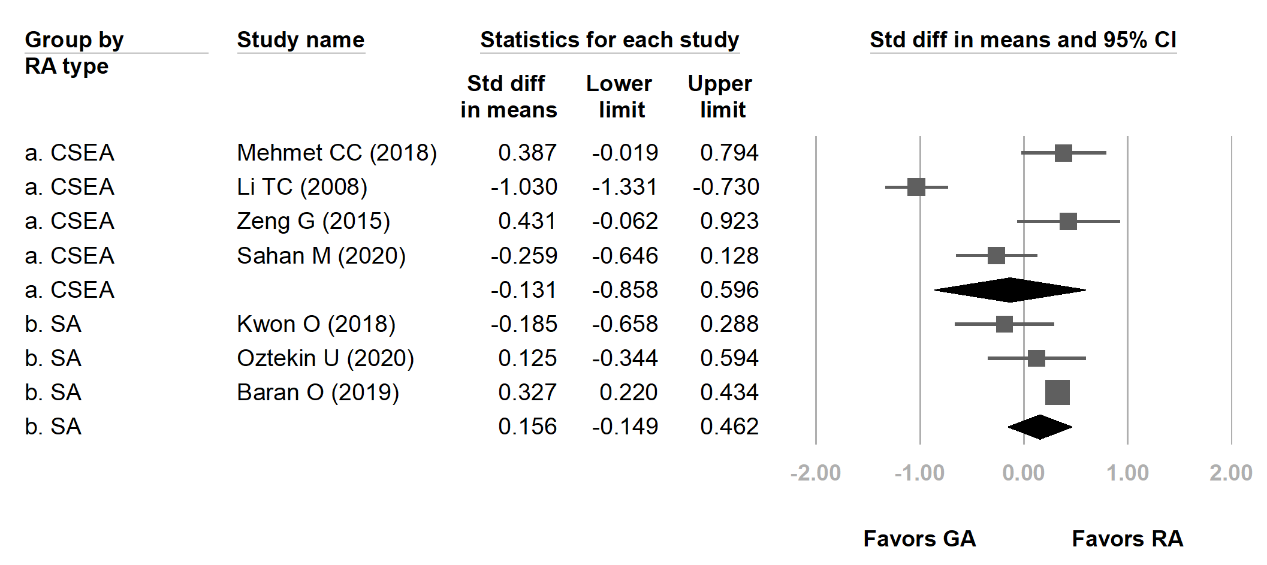


**Supplementary Figure 4.** Subgroup analysis forest plots for the operation time according to RA types (CSEA vs SA)


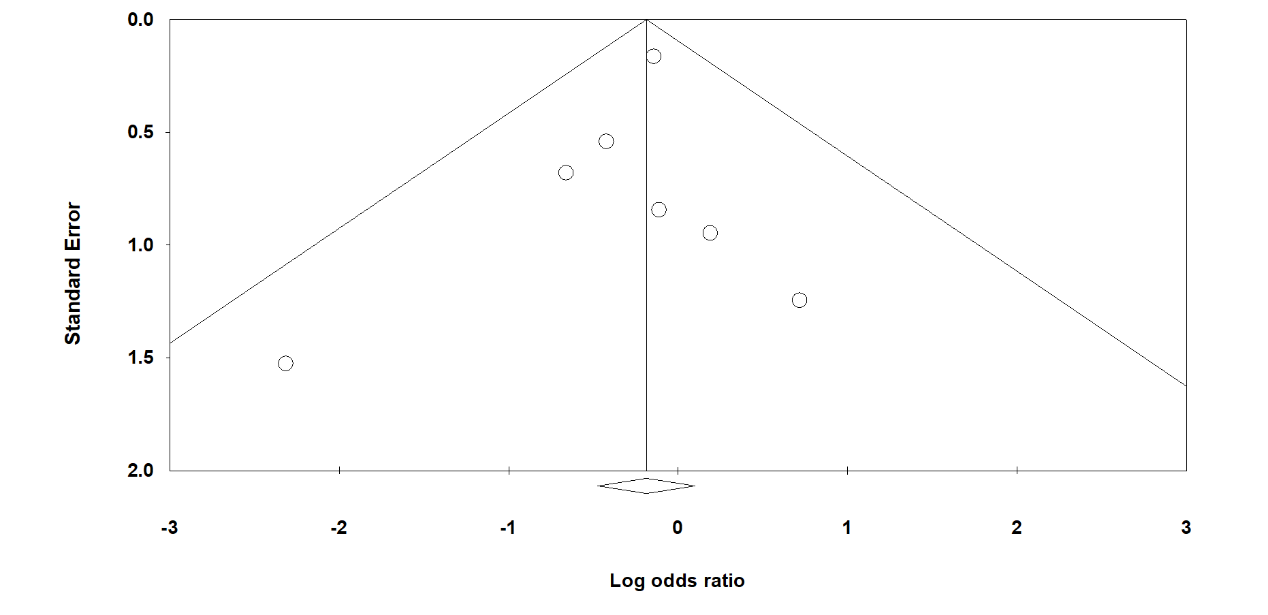


**Supplementary Figure 5.** Funnel plot for the meta-analysis of total complication rates between RA and GA
